# Incidents in Office Practice

**Published:** 1897-06

**Authors:** G. V. N. Relyea

**Affiliations:** Oswego, N. Y.


ARTICLE VIII.

### INCIDENTS IN OFFICE PRACTICE.

BY G. V. N. RELYEA, L. D. S., OSWEGO, N. Y.

Stratagem at times is as necessary in dentistry as in war. A patient called, when the following colloquy ensued: "Some twelve or fourteen years since you filled these front teeth with gold, and they are so nice that I will have nothing but gold in my mouth. My eye tooth is decayed, and if you think it will reach the nerve I will not have it done, but instead will have it extracted. You once killed a nerve for me, and it almost killed me."

I examined the tooth and assured her that it was not very bad, it would not reach the nerve. I commenced to excavate, and doing it carefully had it, as I supposed, ready for the filling. On close examination, however, I saw the pulp shine through the thin wall and at once abandoned the idea of filling with gold. In my palmy days, when I was ambitious, I would have considered this a grand opportunity for capping. In this case there were potent reasons for not adopting it, and in a future article I will give my experience about capping; when and why—when not and why. To tell her the real condition would have involved objections, explanations, etc. In such a case I retire for deliberation, and in this instance came to the conclusion to ask for time. The gums had been somewhat wounded and I advised her to defer the operation and let the gums heal. Then I applied my devitalizer and covered it securely with a four per cent. cocaine. In two days I was to see her again. At the next sitting she reported no pain. On examination I found the pulp devitalized and no inflammation. Quietly and without creating any suspicion I gently removed the pulp almost to the apex, then forced pure wood creasote up and advised a temporary filling, and she could have a gold filling later. When next in the chair I showed her the filling, which was white amalgam, and said why not leave it as it is, it does not show from the outside and it will save you several dollars, to which she gladly consented. I then told her about my destroying the nerve, removing it entire unknown to her, and such a blank, disappointed expression you can scarcely imagine. I have written this for the benefit of young operators.—*Dominion Dental Journal.*

---

ARTICLE IX.

